# Treatment Options in Congenital Disorders of Glycosylation

**DOI:** 10.3389/fgene.2021.735348

**Published:** 2021-09-10

**Authors:** Julien H. Park, Thorsten Marquardt

**Affiliations:** Department of General Pediatrics, Metabolic Diseases, University Children’s Hospital Münster, Münster, Germany

**Keywords:** glycosylation, congenital disorder of glycosylation, treatment, drug repurposing, chaperone, substrate supplementation, cofactor

## Abstract

Despite advances in the identification and diagnosis of congenital disorders of glycosylation (CDG), treatment options remain limited and are often constrained to symptomatic management of disease manifestations. However, recent years have seen significant advances in treatment and novel therapies aimed both at the causative defect and secondary disease manifestations have been transferred from bench to bedside. In this review, we aim to give a detailed overview of the available therapies and rising concepts to treat these ultra-rare diseases.

## Introduction

**FIGURE 1:**
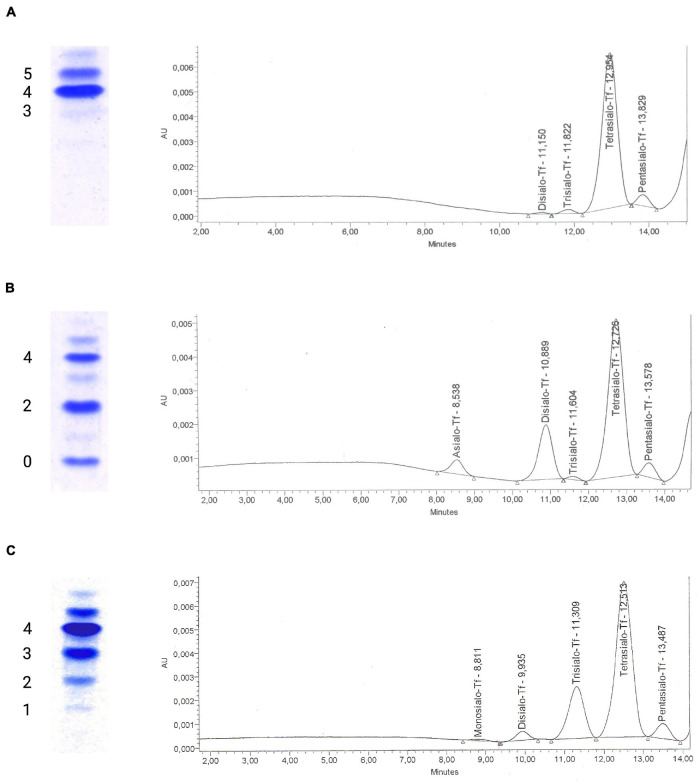
Glycosylation analysis of serum transferrin using isoelectric focusing and high-performance liquid chromatography. Isoelectric focusing (IEF) of serum transferrin has traditionally been used to diagnose congenital disorders of *N*-glycosylation. The test separates transferrin isoforms according to their negative charge that is dependent on the amount of sialic acid residues on glycan chains, with each sialic acid residue corresponding to a negative charge. Currently, alternative methods such as high-performance liquid chromatography (HPLC) are being favored for their ease of use and ability to generate quantitative results for transferrin isoforms. **(A)** A normal glycosylation profile in IEF of serum transferrin with tetrasialo-transferrin (4-) representing the major fraction of transferrin isoforms. To the right, the corresponding HPLC curve can be seen, giving the area % for the varying transferrin subtypes (pentasialo-transferrin 4.97%, tetrasialo-transferrin 92.44%, trisialo-transferrin 1.94%, and disialo-transferrin 0.65%). **(B)** Impaired transferrin glycosylation seen in a PMM2-CDG patient, with decreased tetrasialo-transferrin and increased di-(2-) and monosialo-(1-)transferrin proportions (type I CDG pattern). HPLC identified pentasialo-transferrin (4.62%), tetrasialo-transferrin (68.02%), trisialo-transferrin (0.89%), disialo-transferrin (21.36%), and asialo-transferrin (5.1%). **(C)** In a COG6-CDG patient, increased proportions of tri- (3-), di- (2-), and monosialo-transferrin (1-) are seen in IEF. HPLC detected pentasialo-transferrin (5.2%), tetrasialo-transferrin (69.16%), trisialo-transferrin (22.03%), disialo-transferrin (2.99%), and monosialo-transferrin (0.62%). Reference intervals for HPLC of serum transferrin at our laboratory: Pentasialo (5-) 2.6–10.2%, tetrasialo (4-) 85.7–94.0%, trisialo (3-) 1.16–6.36%, disialo (2-) 0.38–1.82%, monosialo (1-) 0%, asialo (0-) 0%.

Congenital disorders of glycosylation are a group of inborn errors of metabolism affecting the synthesis, processing, and addition of carbohydrate entities to macromolecules, resulting in an extremely varied group of phenotypes affecting multiple organ systems. Initially termed “carbohydrate-deficient glycoprotein syndrome,” disorders of protein *N*-glycosylation were the first to be characterized (Jaeken et al., 1984). Currently, four subgroups of glycosylation disorders are recognized: (A) disorders of *N*-linked glycosylation, (B) disorders of *O*-linked glycosylation, (C) combined *N*- and *O*-linked/multiple disorders of glycosylation, and (D) lipid and glycosylphosphatidylinositol (GPI) anchor biosynthesis defects. Disorders of *N*-glycosylation are subdivided into CDG type I affecting glycan synthesis and type II affecting glycan processing (Marquardt and Denecke, 2003). In the analysis of serum transferrin, the screening method of choice for disorders of *N*-glycosylation, these subtypes are readily distinguished (Figure 1): in type I CDG, di- and asialo-transferrin are elevated, while type II CDG is characterized by more or less inconstantly elevated tri-, di-, mono- and asialo-transferrin (Jaeken and Matthijs, 2001).

The analysis of transferrin glycosylation by isoelectric focusing (IEF), while still being considered the gold-standard, has been replaced by high-performance liquid chromatography (HPLC)- and capillary electrophoresis (CE)-based methods mainly due to the advantages of offering a quantitative assessment of glyco-isoforms and faster turn-around times (Lefeber et al., 2011). More detailed studies of serum transferrin glycosylation can be performed using electrospray ionization quadrupole time-of-flight (ESI QTOF) mass spectrometry of immunopurified serum transferrin (Chen et al., 2019; Wada, 2020). In recent years, mass-spectrometry based analyses of the *N*-glycome, i.e., the entirety of plasma glycan structures, have been shown to detect more subtle glycosylation abnormalities and are being discussed as a first-in-line tool for diagnosing CDG (Wada, 2006; Guillard et al., 2011).

While a plethora of new subtypes has been discovered over the course of the following years and continue to be (Ondruskova et al., 2021), treatment options remained limited to a few subtypes and showed varying success. Recently, the screening of large compound libraries and innovative concepts have identified novel opportunities to treat several subtypes of inborn glycosylation disorders. However, the lack of randomized controlled trials continues to hamper efforts toward a standardized treatment of CDG. In this review, we aim to give a comprehensive overview of past and present approaches to therapy of CDG, stressing rising concepts and recent advances. Special focus is put on treatment approaches to the most common subtype, PMM2-CDG.

## Therapeutic Concepts in the Treatment of CDG

While the ever-growing number of CDG subtypes involves a plethora of disease mechanisms in different organ systems (Jaeken, 2011), the approaches to treating this diverse group of disorders can be summarized by three basic concepts. These have been implemented in clinical care to different degrees with some being firmly established, while others remain preclinical or on a single case basis. In addition, non-specific treatment options are available (Table 1).

**TABLE 1:** Overview and categorization of treatable congenital disorders of glycosylation.

Therapeutic principle	Therapeutic compound	CDG subtype	Phenotype summary
Substrate supplementation	Mannose	MPI-CDG	Liver fibrosis Protein losing enteropathy Coagulopathy Failure to thrive
	Galactose	SLC35A2-CDG	Neurodevelopmental delay Seizures Brain malformations Dysmorphic features Skeletal abnormalities
		PGM1-CDG	Myopathy Cardiomyopathy Hepatopathy Hypoglycemia Dysmorphic features Endocrinopathies
		SLC39A8-CDG*	Neurodevelopmental delay Seizures Cerebellar atrophy Cranial synostoses Visual impairment Auditory impairment Failure to thrive Leigh-like syndrome
		TMEM165-CDG*	Neurodevelopmental delay Skeletal dysplasia Hepatopathy Nephrotic syndrome Cardiac defects
	Fucose	SLC35C1-CDG	Neurodevelopmental delay Short stature Facial dysmorphism Recurring infections
		FUT8-CDG	
Cofactor supplementation	Manganese-(II)-sulfate	SLC39A8-CDG	See above
Pharmaceutical chaperones	Epalrestat	PMM2-CDG	See above
Non-causative and other treatments	Acetazolamide	PMM2-CDG	See above
	Sodium butyrate	PIGM-CDG	Seizures Thrombotic events Muscular hypotonia Macrocephaly

*Galactose supplementation in these disorders targets secondary glycosylation abnormalities (see section “Galactose Supplementation Corrects Secondary Glycosylation Abnormalities in SLC39A8-CDG and TMEM165-CDG”).

### Substrate (Precursor) Supplementation and Bypassing Strategies

**FIGURE 2:**
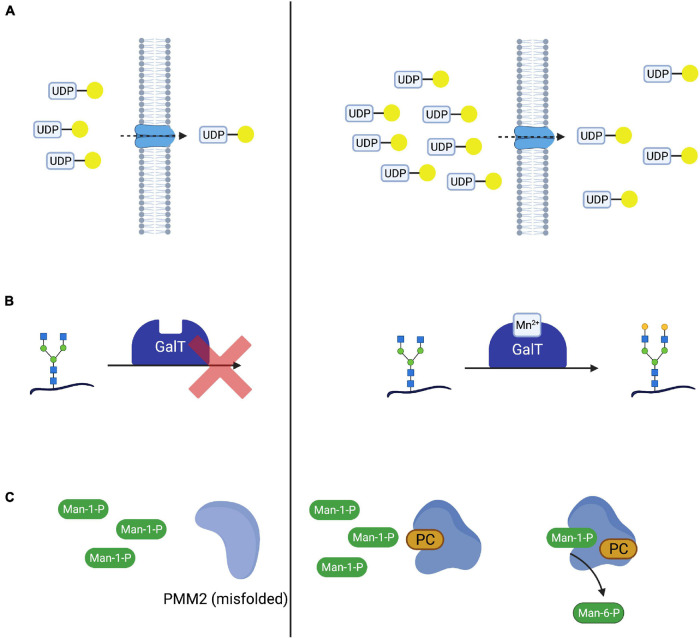
Therapeutic concepts for congenital disorders of glycosylation. **(A)** Substrate supplementation aims at overcoming the impaired transport process or enzymatic reaction by increasing the concentration of the substrate of the respective reaction. One such treatment is galactose supplementation for SLC35A2-CDG, where oral supplementation of galactose (yellow circle) increases UDP-galactose supplies and thus transport across the defective UDP-galactose transporter SLC35A2. **(B)** In SLC39A8-CDG, defects in SLC39A8 lead to a deficiency in manganese (Mn^2+^). Lack of this cofactor impairs the function of galactosyltransferases (GalT). Cofactor supplementation leads to an improved GalT function and thus normalized glycosylation. **(C)** Mutated and subsequentially misfolded enzymes are either degraded or impaired in function. Specific mutations in *PMM2* result in misfolded phosphomannomutase 2 **(A)**. Pharmacological chaperones (in this case epalrestat) bind and stabilize the affected enzyme, leading to increased enzyme activity and thus improved glycosylation **(B)**.

With the elucidation of underlying enzymatic defects, initial attempts were made to supplement substrates of the affected enzymes with the aim to shift the reaction equilibrium toward the favored product, thus improving glycosylation. In cases where the direct substrate is either not available or nor stable, precursors of this substrate have been applied or the enzymatic defect was bypassed, utilizing alternative pathways (see section “Mannose Supplementation for MPI-CDG Bypasses the Impaired Enzyme”). In analogy to this, the transported molecule (“substrate”) of transport proteins has been supplemented in CDG subtypes mediated by nucleotide sugar transporter defects (Figure 2A; Eisenberg, 2011). While successful in several CDG subtypes such as MPI-CDG, SLC35C1-CDG, and SLC35A2-CDG (Niehues et al., 1998; Marquardt et al., 1999; Witters et al., 2020), the therapeutic principle remains disputed in the most common subtype PMM2-CDG.

Most proposed substrate supplementation therapies have been administered orally and are therefore sometimes considered to be nutritional therapies (Verheijen et al., 2020). Attempts at parenteral, i.e., intravenous, substrate supplementation have been made (Mayatepek et al., 1997; Schroeder et al., 2010; Grünert et al., 2019) but have typically been limited to critically ill patients not tolerating oral supplementation and were either unsuccessful (i.v., mannose in PMM2-CDG) or associated with adverse effects (i.v., mannose in MPI-CDG, see section “Mannose Supplementation for MPI-CDG Bypasses the Impaired Enzyme”).

### Cofactor Supplementation

In several CDG subtypes, supplementation of the affected enzyme with essential cofactors has been employed as a means to improve glycosylation both *in vivo* and *in vitro* (Park et al., 2018; Houdou et al., 2019). Like substrate supplementation, the addition of cofactor(s) aims at improving protein function by optimizing reaction conditions in order to shift the reaction equilibrium toward the product (Figure 2B). An emerging subgroup of glycosylation disorders is caused by genetic defects affecting the uptake of cofactors with glycosylation abnormalities as a secondary, “downstream” manifestation of the disorder. In these CDG, cofactor supplementation is a promising therapeutic concept that has been established for some of these (Potelle et al., 2017; Park et al., 2018).

### Pharmacological Chaperones

While frameshift and non-sense mutations frequently lead to a total loss of protein function (Gorlov et al., 2006), missense mutations can result in impaired protein folding (Yuste-Checa et al., 2015) and thus reduced enzyme activity. Pharmacological chaperones are small molecules capable of binding to the altered structure of mutated proteins and facilitating correct folding and thus increasing enzyme activity (Figure 2C). This principle has been explored in lysosomal storage disease and there are currently approved medications for Fabry disease (Fan et al., 1999; Germain et al., 2016) and Niemann-Pick C disease (Pipalia et al., 2019, 2021; Shioi et al., 2020), while such treatments for others are under investigation (Tropak et al., 2004; Parenti et al., 2014; Han et al., 2020). The advent of *in silico* screening of large compound libraries has greatly facilitated the identification of novel candidate compounds and is actively being explored in CDG (Yuste-Checa et al., 2016).

### Non-causative and Other Treatments

Besides treatments aiming at correcting or improving the function of the affected protein and thus leading to normalized glycosylation, other treatments aim to correct symptoms or secondary manifestations of the disease. In the interest of brevity, only those treatments specific to symptoms of CDG are mentioned in this review.

Furthermore, the treatment of glycosylphosphatidylinositol (GPI) deficiency caused by promoter mutations in *PIGM* relies on enhanced histone acetylation, a concept not used in any other known CDG therapy (Almeida et al., 2007).

## Established Therapies for Congenital Disorders of Glycosylation

### Substrate (Precursor) Supplementation and Bypassing Strategies

#### Mannose Supplementation for MPI-CDG Bypasses the Impaired Enzyme

**FIGURE 3:**
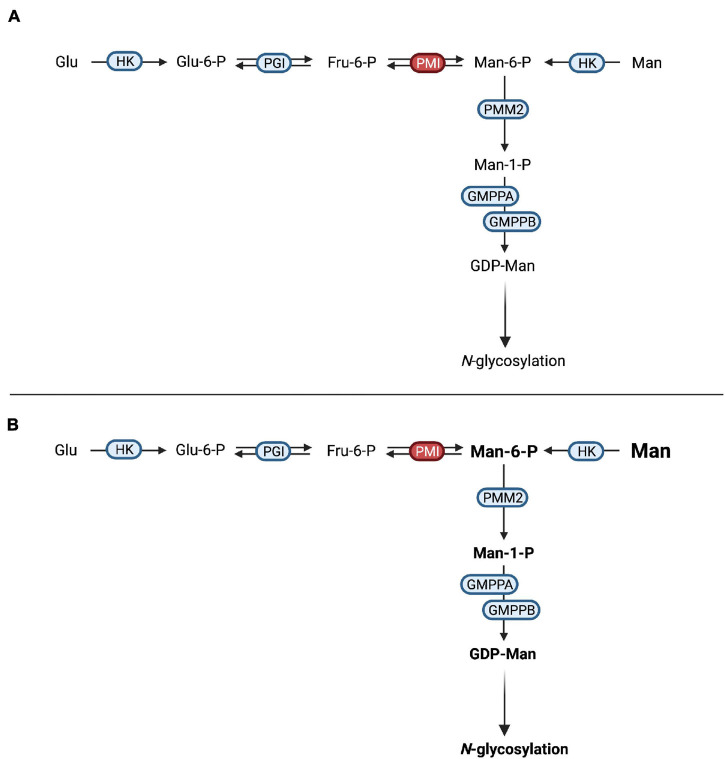
Mannose therapy in MPI-CDG. **(A)** Mutations in *MPI* lead to an impaired function of phosphomannose isomerase (PMI), thus hindering the interconversion of fructose-6-phosphate (Fru-6-P) to mannose-6-phosphate (Man-6-P). **(B)** The oral supplementation of mannose increases the available Man-6-P following conversion of mannose (Man) by hexokinase (HK). After conversion of Man-6-P to mannose-1-phosphate (Man-1-P) by phosphomannomutase 2 (PMM2), conversion into guanosine diphosphate-mannose (GDP-Man). This can be used in *N*-glycosylation.

Defects in mannose-6-phosphate isomerase (PMI, EC 5.3.1.8) cause MPI-CDG (Figure 3A), a disorder characterized by severe protein-losing enteropathy (Jaeken et al., 1998; Niehues et al., 1998), liver disease (de Koning et al., 1999), and coagulopathy resulting in recurrent thrombosis (Girard et al., 2020). In addition, hyperinsulinism occurs frequently (de Lonlay et al., 1999). Unlike in other CDG, no neurological phenotype can be observed and patients show no intellectual disabilities (de Lonlay and Seta, 2009).

Due to the loss of PMI function, the conversion of fructose-6-phosphate into mannose-6-phosphate is not possible, necessitating the direct phosphorylation of mannose by hexokinase (Niehues et al., 1998). Since the mammalian mannose transporter was found to operate at submaximal efficiency under physiological mannose concentrations (Etchison and Freeze, 1997; Panneerselvam et al., 1997) and oral incorporation of mannose was shown to raise blood levels (Alton et al., 1997), oral mannose supplementation was introduced and showed both clinical and biochemical improvement in a follow-up period of 11 months (Niehues et al., 1998; Figure 3B). Additional studies showed similar results (Mayatepek and Kohlmüller, 1998; de Lonlay et al., 1999; Westphal et al., 2000; Hendriksz et al., 2001; Harms et al., 2002) with improvement of intestinal and hematological symptoms, while the response of liver disease to mannose therapy might be limited (de Lonlay and Seta, 2009). Interestingly, hepatic fibrosis may be present at birth, hinting at a ante-natal onset of liver disease that might not be amenable to postnatal mannose treatment (Girard et al., 2020).

Although these results were convincing with regards to efficacy, the observed toxicity of mannose in several *Apidae* dubbed “honeybee syndrome” (Sols et al., 1960) raised concerns regarding the safety of this intervention. Mannose toxicity in these insects was found to be caused by intrinsic MPI deficiency (Sols et al., 1960) and subsequent accumulation of mannose-6-phosphate in conjunction with intracellular ATP depletion (de la Fuente et al., 1986).

In the context of mannose therapy for MPI-CDG, oral substitution is usually well tolerated but intravenous administration of mannose in a patient was associated with central nervous and hepatic dysfunction that was reversible upon increased intravenous glucose substitution (Schroeder et al., 2010). This was attributed to intracellular ATP depletion in addition to inhibited glycolysis by mannose-6-phosphate, similar to findings from animal model (DeRossi et al., 2006).

These findings culminated in the recent proposition of international consensus guidelines recommending the oral administration of mannose at a concentration of 150–170 mg/kg bodyweight four to five times per day (Čechová et al., 2020), a treatment that has been approved in both the European Union (EU) and the United States (Brasil et al., 2018). Blood mannose levels can serve to monitor treatment for dose optimization with measurements before administration and after 1 h, aiming to achieve levels of >20 and >100 μmol/L, respectively (Čechová et al., 2020). Despite the high efficacy of treatment regarding intestinal and hematological symptoms, the requirement to ingest large amounts of mannose and associated adverse effects such as diarrhea may lead to poor compliance (Girard et al., 2020). Future improvements in formulation might improve therapy adherence.

In addition to mannose, heparin has shown positive effects on protein losing enteropathy associated with MPI-CDG in a single case (Liem et al., 2008).

#### Galactose Supplementation Improves Defective UDP-Galactose Transport in SLC35A2-CDG

Mutations in *SLC35A2* affecting the function of the Golgi-localized UDP-galactose transporter (Figure 2A) are inherited in an X-linked recessive manner although most occur *de novo*, resulting in a type II CDG (Kodera et al., 2013; Ng et al., 2013). In some cases, glycan analysis indicates no abnormal glycosylation of serum transferrin (Ng et al., 2019). The phenotype is characterized by seizures often manifesting as severe infantile spasms with hypsarrhythmia, failure to thrive, dysmorphic features, and brain malformations.

Glycan analysis indicates a loss of both galactose and sialic acid structures and *in vitro* studies have shown reduced uptake and subsequently a severely reduced Golgi-localized UDP-galactose following the expression of *SLC35A2* mutations (Ng et al., 2013). On the background of these findings, oral galactose supplementation at doses of up to 1.5 g/kg bodyweight/day or higher was proposed as a potential treatment and correlated with normalized transferrin glycosylation as well as clinical improvement (Dörre et al., 2015). However, improvement of transferrin glycosylation was also observed in untreated individuals (Ng et al., 2019). On the background of the absence of dysglycosylation in a group of affected individuals, improved glycosylation can – in our view – be seen as an unreliable correlate for treatment efficacy at best. A recently published study underscored positive effects on clinical presentation, namely seizure control, as well as biochemical abnormalities, further strengthening the case for galactose supplementation as a treatment for SLC35A2-CDG (Witters et al., 2020).

#### Rewiring Glucose Metabolism and Glycosylation – Galactose Supplementation in PGM1-CDG

Before their identification as a cause of a glycosylation disorder in 2014 (Tegtmeyer et al., 2014), biallelic mutations in *PGM1* were identified as a cause of glycogen storage disease (GSD) type XIV (Stojkovic et al., 2009). In the index patient, rhabdomyolysis and muscle weakness along muscular glycogen accumulation were noted while PGM1 activity was severely reduced, leading to decreased interconversion of glucose-1-phosphate and glucose-6-phosphate.

**FIGURE 4:**
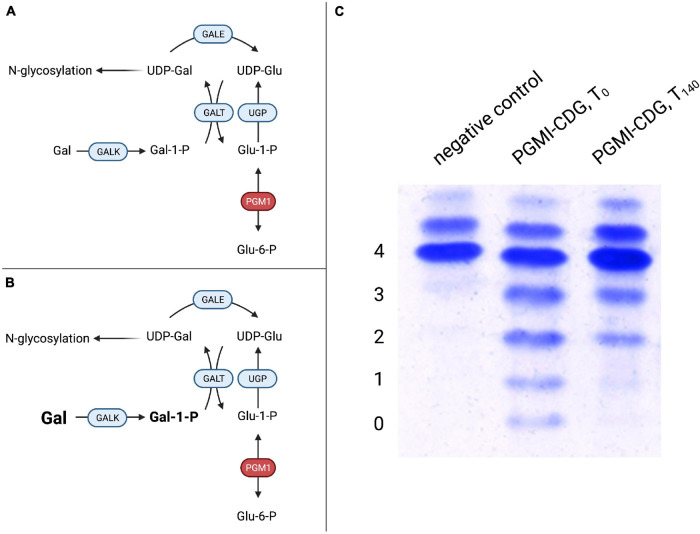
Galactose therapy in PGM1-CDG. **(A)** A defect in PGM1 hinders conversion of glucose-1-phosphate (Glu-1-P) to glucose-6-phosphate (Glu-6-P), thus impairing hepatic glucose release. Similarly, the reverse reaction in which Glu-6-P is converted to Glu-1-P, which can serve as a substrate for UDP-glucose production by UDP-glucose pyrophosphorylase (UGP) for glycogen synthesis or glycosylation, is impaired. **(B)** Supplemented galactose is transformed to UDP-galactose (UDP-Gal) by galactose-1-phosphate uridyltransferase (GALT). UDP-Gal can serve both in glycosylation and as a substrate for UDP-galactose epimerase (GALE), supplying UDP-Gal for glycogen metabolism. **(C)** Transferrin IEF profiles during galactose substitution over 140 days in PGM1-CDG. Compared to controls, PGM1-CDG shows a characteristic, “ladder-like” pattern in IEF (PGM1-CDG, T_0_). Following 20 weeks of galactose substitution at a dose of 1 g/kg bodyweight/day, significant improvement with reduced abnormal transferrin isoforms was observed (PGM1-CDG, T_140_) (4 – tetrasialo-transferrin, 3 – trisialo-transferrin, 2 – disialo-transferrin, 1 – monosialo-transferrin, 0 – asialo-transferrin).

Subsequently, a large cohort of 19 patients with biallelic *PGM1* mutations leading to abnormal glycosylation with a mixed type I and II like dysglycosylation pattern of serum transferrin was identified (Tegtmeyer et al., 2014). Additional phenotypical features included hypoglycemia, hepatopathy (transaminase elevation, abnormal coagulation parameters), growth retardation, and dilated cardiomyopathy. Endocrine abnormalities in the form of hypogonadotropic hypogonadism were present, while a bifid uvula was described as an easily recognizable clinical sign. Initially, it was believed that neurological impairment was uncommon and secondary to hypoglycemia. However, recent studies have proposed a neurological phenotype unrelated to blood sugar abnormalities (Radenkovic et al., 2018). Due to the finding of markedly decreased UDP-galactose content in patient-derived skin fibroblasts, galactose was added to the cell culture media and resulted in improved glycosylation of ICAM-1 while no effect on glycogen content was observed. This intervention does not provide the direct substrate of PGM1 but bypasses the enzymatic defect (Figures 4A,B), resulting in improved glycosylation (Figure 4C).

The subsequent galactose supplementation in a sub-cohort of six individuals of 1 g/kg bodyweight/day led to improved glycosylation as assessed by serum transferrin and total serum *N*-glycome studies. Clinically, no further episodes of rhabdomyolysis were observed and hypogonadotropic hypogonadism resolved with patients developing signs of puberty (Tegtmeyer et al., 2014).

A prospective trial in eight patients confirmed the positive effect observed previously (Wong et al., 2017). In the study, D-galactose was administered orally at incremental doses of 0.5, 1.0, and 1.5 g/kg/day for 6 weeks, respectively (total study period 18 weeks). One individual continued to receive a lesser dose of 1.0 g/kg/day for a year after the trial. The absence of serious adverse events and generally good tolerance of increasing amounts of galactose indicated the general safety of the therapy. As in the previous study, transferrin glycosylation improved in all except one participant, and no further episodes of rhabdomyolysis were reported despite no clear effect on previously elevated creatine kinase levels. Endocrine abnormalities improved in all patients. In addition, liver function improved drastically, with ALT normalizing in a subset of patients and AST decreasing. Likewise, coagulation parameters improved or normalized. In a separate trial with eleven individuals, early therapy was found to be preferrable to delayed treatment, which was seen to argue for inclusion into screening programs (Conte et al., 2020). This is especially relevant since a modified Beutler test was shown to detect PGM1-CDG from dried blood spots (Tegtmeyer et al., 2014). A glycoprofiling study was able to identify specific glycomarkers, allowing early diagnosis as well as therapy monitoring using mass spectrometry-based methods (Abu Bakar et al., 2018).

The effect of galactose supplementation on cardiomyopathy has not been evaluated formally so far. In a singular case that presented with restrictive rather than dilated cardiomyopathy, galactose supplementation did not improve echocardiography and ECG results, while liver function and glycosylation improved (Donoghue et al., 2021). Similarly, there are reports of incomplete normalization of transferrin glycosylation at standard doses of galactose, possibly indicating that supplementation at higher doses as done, e.g., in SLC39A8-CDG might be needed in certain cases (Nolting et al., 2017).

#### Galactose Supplementation Corrects Secondary Glycosylation Abnormalities in SLC39A8-CDG and TMEM165-CDG

Both SLC39A8-CDG and TMEM165-CDG are caused by disturbed manganese metabolism and might thus be considered secondary glycosylation disorders in which the deficiency of glycosyltransferases is caused by the lack of a cofactor (see section “Manganese-Sulfate Is a Causative Treatment for SLC39A8-CDG” and “A Potential Role for Manganese in the Treatment of TMEM165-CDG,” Figure 2B). Mass spectrometry analysis of glycan structures has identified hypogalactosylation, i.e., the lack of galactose residues when compared to normal glycan structures, in both subtypes (Foulquier et al., 2012; Park et al., 2015).

Oral supplementation of galactose has therefore been attempted in order to improve dysglycosylation. In SLC39A8-CDG, a dose of up to 3.75 g/kg bodyweight per day (either given continuously via an enteral feeding pump or divided in five equal doses) led to near complete normalization transferrin glycosylation, while interruption of treatment resulted in an increase of abnormally glycosylated transferrin isoforms (Park et al., 2015). The relatively high doses used here were well tolerated, hinting at the possibility of higher doses in other CDG subtypes (see section “Galactose Supplementation Improves Defective UDP-Galactose Transport in SLC35A2-CDG” and “Rewiring Glucose Metabolism and Glycosylation – Galactose Supplementation in PGM1-CDG”). In addition to galactose, uridine was supplemented (150 mg/kg bodyweight/d) with the aim to increase UDP-galactose supplies. The trial design did not allow for a distinction between the possible separate effects of galactose and uridine. Similar results were seen in an independent study (Riley et al., 2017). In both cases, improvement was observed within approximately 2 weeks, indicating that transferrin synthesis might be the limiting factor (Park et al., 2015).

Similarly, galactose supplementation was studied in TMEM165-CDG. After encouraging results on both a HEK293 model with a knockout of *TMEM165* and patient-derived skin fibroblasts in which galactose corrected hypogalactosylation (Morelle et al., 2017), oral supplementation of 1 g galactose/kg/d was administered to two individuals with TMEM165-CDG. *N*-glycosylation improved and biochemical abnormalities were partly corrected, with higher doses of up to 1.5 g/kg/d having no additional beneficial effect.

#### L-Fucose Supplementation Increases Impaired GDP-Fucose Transport in SLC35C1-CDG

Leukocyte adhesion deficiency type II (LADII) is caused by impaired glycoconjugate fucosylation due to impaired function of SLC35C1, the GDP-fucose transporter, and is therefore named SLC35C1-CDG under the current nomenclature of glycosylation disorders. Even before the identification of the underlying genetic defect (Lübke et al., 2001; Lühn et al., 2001), reduced fucose uptake and correction by increased fucose supplementation both *in vitro* (Lübke et al., 1999; Marquardt et al., 1999) and *in vivo* (Marquardt et al., 1999; Wild et al., 2002) was demonstrated.

The phenotype is marked by severely delayed psychomotor development in conjunction with short stature, dysmorphic features, and recurrent, potentially life-threatening infections (Etzioni et al., 1992). Extreme neutrophilia (up to 20 times of normal values) due to impaired rolling is caused by the absence of sialyl-Lewis^*x*^ (sLe^*x*^, CD15) selectin ligand carrying fucose (Etzioni et al., 1992). Similarly, patients have a Bombay blood group (hh) as defined by lacking expression of the H antigen with an alpha(1,2)linked fucose-galactose disaccharide (Wolach et al., 2019). In a subset of patients, the immunological phenotype appears to be rather mild and these individuals are frequently diagnosed with short stature and intellectual disability (Dauber et al., 2014; Knapp et al., 2020).

Oral L-fucose supplementation has been administered five times per day in escalating doses of up to 492 mg/kg bodyweight/dose and was shown to correct core fucosylation of serum proteins, followed by a reduction of peripheral neutrophil counts (Marquardt et al., 1999). During therapy, improvement of psychomotor development as assessed by the Griffiths Test (Griffiths, 1984) was observed, although no standardized trials regarding psychomotor development during fucose therapy of SLC35C1-CDG have been conducted. The involvement of blood group antigens in the phenotype necessitates careful observation during therapy: In theory, synthesis of the H-antigen might occur during fucose supplementation, causing autoimmune side effects in case anti-H-antigen antibodies are present or would be raised. However, no H-antigen expression has been observed in the original trial (Marquardt et al., 1999) or has occurred to our knowledge.

#### L-Fucose Supplementation Leads to Clinical Improvement and Protein-Specific Enhancement of Glycosylation in FUT8-CDG

Mutations in *FUT8* encoding the α-1,6-fucosyltransferase (EC 2.4.1.68) are associated with a severe glycosylation disorder that is characterized by a loss of core fucosylation upon glycan analysis in patient sera as well as patient-derived cells (Ng et al., 2018). All patients exhibit failure to thrive, severe developmental delay, muscular hypotonia, feeding abnormalities, with respiratory abnormalities being a distinctive feature of the disorder (Ng et al., 2020). Seizures are also frequently seen, although absence of this disease manifestation has been observed (Park et al., 2020b).

The addition of L-fucose to cell culture media of patient-derived skin fibroblasts did not improve either overall glycosylation or core fucosylation in a specific variant leading to a loss of exon 9 of the protein (Ng et al., 2018). In contrast, oral L-fucose supplementation in dizygotic twins that was escalated from 100 to 825 mg/kg/d showed a protein specific effect with increased core fucosylation of both serum transferrin and IgG in addition to an increase of a fucosylated disialo-biantennary glycan. At the same time, several truncated, non-fucosylated glycan entities (agalactosylated glycan Hex3HexNAc4, asialo glycan Hex5HexNAc4, and mono-galactosylated, Neu5Ac1Hex4HexNAc4) decreased to normal levels (Park et al., 2020b). No adverse effects were noted during follow-up and the patients could be weaned from non-invasive ventilation and showed general clinical improvement.

#### Sialic Acid Supplementation Is Associated With Improved Psychomotor Development in NANS-CDG

First described in 2016, NANS-CDG is caused mutations in the eponymous gene, leading to impaired function of *N*-acetyl-*D*-neuraminic acid synthase (van Karnebeek et al., 2016). Phenotypically, the disorder is characterized by global developmental delay, muscular hypotonia, short stature, and facial dysmorphisms. In a zebrafish model, sialic acid was able to partially rescue the phenotype (van Karnebeek et al., 2016). Early results indicate improved psychomotor development in NANS-CDG patients following sialic acid supplementation (den Hollander et al., 2021), with additional research still ongoing.

#### Impaired Nucleotide Sugar Synthesis in CAD-CDG Is Rescued by Uridine Supplementation

Mutations in *CAD* lead to a deficiency in cytoplasmic carbamoyl-phosphate synthetase 2, subsequently impairing *de novo* pyrimidine synthesis. Loss of the protein results in a reduction of nucleotide sugars, i.e., precursors of glycosylation, and reduced flux of aspartate into DNA and RNA (Ng et al., 2015). Of note, despite reduced nucleotide sugars, no abnormal glycosylation was found in fibroblasts or serum of the index patient. This was confirmed in an additional cohort of two patients (Koch et al., 2017), making the classification of CAD deficiency as a glycosylation disorder debatable. Clinically, affected individuals show global developmental delay, epileptic encephalopathy and a hematologic phenotype consisting of anemia and anisopoikilocytosis (Ng et al., 2015; Koch et al., 2017). After rescue of biochemical abnormalities *in vitro* by uridine supplementation (Ng et al., 2015), a therapeutic trial was initiated resulting in cessation of seizures as well as normalization of biochemical abnormalities (Koch et al., 2017).

#### Mannose Supplementation in PMM2-CDG?

PMM2-CDG was the first glycosylation disorder and was characterized in the 1980s by the Belgian Pediatrician Jaak Jaeken (Jaeken et al., 1984). Caused by a defect in the enzyme phosphomannomutase 2 (EC: 5.4.2.8), this disturbance of mannose metabolism is an archetypical glycosylation disorder and remains the most common one, with approximately 1,000 cases diagnosed to date (Witters et al., 2018).

**FIGURE 5:**
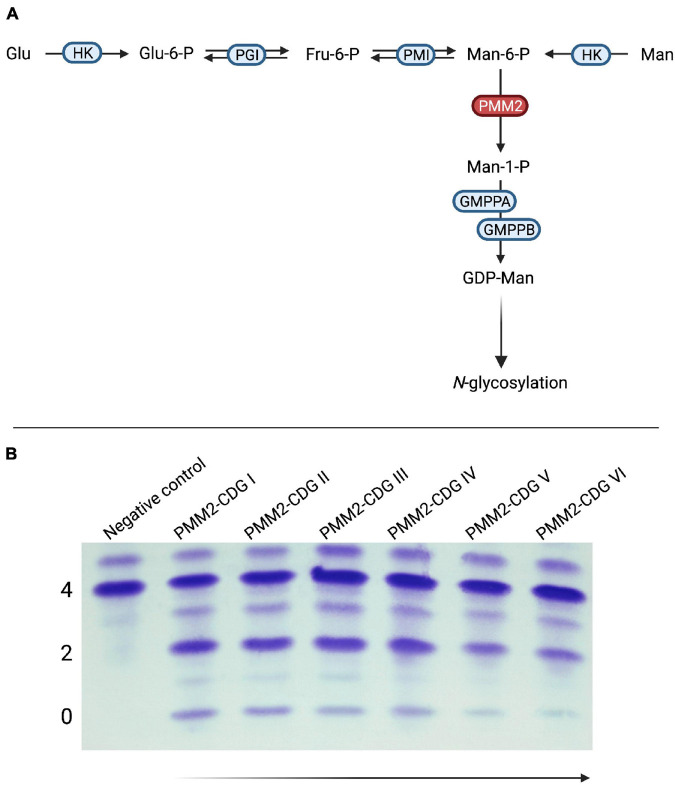
Mannose supplementation in PMM2-CDG. **(A)** In PMM2-CDG, the conversion of mannose-6-phosphate (Man-6-P) to mannose-1-phosphate (Man-1-P) by phosphomannomutase 2 (PMM2) is impaired due to mutations in *PMM2.* **(B)** Findings for mannose supplementation in PMM2-CDG have been inconsistent. In a subgroup of patients, significant improvement of serum transferrin glycosylation can be achieved with oral supplementation of mannose. Of note, these changes occur after several months or even years and have been shown to be reversible if mannose substitution is discontinued. PMM2-CDG I and II – Pretherapeutic samples from the same patient; PMM2-CDG III–VI – Samples after 1, 2, 3, and 4 years of mannose substitution at a dose of 1 g/kg bodyweight/day (HK, hexokinase; Glu-6-P, glucose-6-phosphate; PGI, glucose-6-phosphate isomerase; Fru-6-P, fructose-6-phosphate; PMI, phosphomannose isomerase; GMPPA, Mannose-1-phosphate guanyltransferase alpha; GMPPB, Mannose-1-phosphate guanyltransferase beta).

Early on, mannose supplementation was considered as a possible therapy. However, the location of the enzyme defect within the glycosylation pathway renders a beneficial effect of exogenous mannose unlikely (Figure 5; Ichikawa et al., 2014). Surprisingly, treatment with mannose led to a normalization of glycosylation in patient-derived skin fibroblasts (Panneerselvam and Freeze, 1996; Rush et al., 2000). Similarly, mannose administration to pregnant mice mitigated embryonic lethality in a hypomorphic PMM2 mouse model (Schneider et al., 2011).

Early trials involving both intravenous (Mayatepek et al., 1997) and oral (Kjaergaard et al., 1998) mannose administration did not show any signs of improvement. These findings were supported by later, unrelated reports (Mayatepek and Kohlmüller, 1998; Grünert et al., 2019). In a recent retrospective analysis of longer mannose supplementation over several years, our group detected biochemical improvement as defined by improved transferrin glycosylation following treatment >1 year (Taday et al., 2020; Figure 5B). Although spontaneous improvement of serum transferrin glycosylation is frequently seen in PMM2-CDG (Schiff et al., 2017; Witters et al., 2018), the response regardless of age at onset of therapy and the deterioration following discontinuation of treatment argue against purely spontaneous normalization (Taday et al., 2020). Due to the retrospective nature of the study, no formal evaluation of clinical improvement was performed. However, a subset of responders showed improved nerve conduction velocities in addition to restoration of deep tendon reflexes (Taday et al., 2020). Therefore, further studies – ideally in the form of randomized-controlled trials – are needed to assess the effect of oral mannose supplementation on PMM2-CDG.

### Cofactor Supplementation

#### Manganese-Sulfate Is a Causative Treatment for SLC39A8-CDG

In contrast to other glycosylation disorders, SLC39A8-CDG is caused by mutations in the gene encoding the eponymous divalent cation channel, that acts as the principal cellular manganese uptake transporter (He et al., 2006; Nebert and Liu, 2019). Due to the reliance of several glycosyltransferases on Mn^2+^ as a cofactor (Breton et al., 2006), mutations in *SLC39A8* resulting in intracellular manganese depletion cause secondary glycosylation defects corresponding to a type II CDG pattern of transferrin dysglycosylation. Manganese is severely reduced or absent both in blood and urine (Boycott et al., 2015; Park et al., 2015; Riley et al., 2017).

Clinically, SLC39A8-CDG is characterized by psychomotor retardation, short stature, severe seizures, cerebellar atrophy, cranial synostoses, as well as visual and auditory impairment (Boycott et al., 2015; Park et al., 2015). In addition, Leigh-like mitochondrial disease, possibly due to impaired function of manganese-dependent SOD2, has been reported (Riley et al., 2017). Indeed, additional manganese dependent enzymes might in theory be affected by manganese depletion.

While initial attempts using galactose supplementation aimed at correcting the observed hypogalactosylation and resulted in improved transferrin glycosylation (Park et al., 2015), manganese supplementation was hypothesized to be a causative treatment not only targeting impaired glycosyltransferases but also other manganese dependent enzymes like SOD2 (Flynn and Melov, 2013) and xanthine oxidase (Schroeder et al., 1966). In a trial with two individuals, oral supplementation of 15–20 mg manganese-II-sulfate (MnSO_4_.H_2_O)/kg bodyweight was able to correct transferrin glycosylation to normal levels and led to considerable clinical improvement by near normalization of EEG patterns and cessation of seizures (Park et al., 2018). However, due to the broad spectrum of clinical presentations, the doses needed for correction cannot be expected to be uniform in all patients.

Given the potential toxicity of manganese, showcased by cases of a Parkinsonian phenotype dubbed “manganism” observed in battery or steel factory workers as well as other occupational or environmental exposition (Harischandra et al., 2019; Evans and Masullo, 2020), caution is warranted in treating SLC39A8-CDG. Although no reports of adverse effects of manganese substitution exist, careful monitoring of blood manganese levels and repeated MRI studies to assess possible manganese deposits in the brain (Lucchini et al., 2000; Crossgrove and Zheng, 2004) seems prudent.

Another hindrance is the imperfect assessment of treatment efficacy. While blood manganese levels and transferrin glycosylation normalize quickly under adequate substitution (Park et al., 2018), recent research by our group has identified subtle glycosylation abnormalities in SLC39A8-CDG that are not detected by conventional methods (Park et al., 2020a). *N*-glycome profiling using matrix-assisted laser desorption ionization time of flight (MALDI-TOF) mass spectrometry might be more suitable to monitor the effects of manganese sulfate substitution (Park et al., 2020a).

#### A Potential Role for Manganese in the Treatment of TMEM165-CDG

As in SLC39A8-CDG, glycosylation defects in TMEM165-CDG are characterized by hypogalactosylation in addition to hyposialylation (Foulquier et al., 2012). Recent studies have identified abnormal manganese metabolism (Potelle et al., 2016, 2017) and provide *in vitro* evidence that manganese can correct glycosylation abnormalities in TMEM165-CDG. To date, no *in vivo* studies have been performed to assess manganese supplementation as a therapy for TMEM165-CDG.

### Chaperones

While having been explored for some time, pharmacological chaperones did not reach clinical application or *in vivo* trials in CDG until recently. However, previous research indicated that glucose-1,6-bisphosphate is a natural ligand of PMM2 and increases its catalytic activity (Monticelli et al., 2019). Following screening studies on large compound libraries (Yuste-Checa et al., 2016), the aldolase inhibitor epalrestat was identified as a potent activator of several variant PMM2 proteins (Iyer et al., 2019). The effect on mutant PMM2 proteins carrying the frequent variants p.R141H/p.F182S, p.R141H/p.E139K, and p.R141H/p.N216I was assessed using a novel *Caenorhabditis elegans* model and patient-derived fibroblasts. An increase in PMM2 activity of up to 3.15-fold of baseline was observed (Iyer et al., 2019).

Due to the compounds approval as a drug for diabetic neuropathy in Japan, clinical application in CDG would represent a repurposing approach with the potential to shorted time to clinical application [for an excellent review on drug repurposing see Pushpakom et al. (2019)]. Currently, a *n* = 1 exploratory study is being conducted in the United States, with results expected to be reported shortly (A Phase I study of Epalrestat Therapy in a Single Patient with Phosphomannomutase Deficiency (PMM2-CDG), 2021). Another patient was started in Germany even earlier and is still under investigation.

### Non-causative and Other Treatments

#### General Aspects and Clinical Recommendations for the Treatment of CDG

General recommendations for the symptomatic treatment of CDG have mainly been based on clinical experience or single case studies rather than controlled trials. A detailed summary on current specific recommendations for PMM2-CDG, MPI-CDG, and PGM1-CDG can be found in published consensus guidelines (Altassan et al., 2019, 2021; Čechová et al., 2020). However, on the background of lacking guidelines for most subtypes, general recommendations that apply to all known CDG subtypes can facilitate management of such patients.

In general, fever exerts a detrimental effect on glycosylation and has been shown to reduce the residual activity of glycosylation related enzymes further (Kjaergaard et al., 1999; Andreotti et al., 2015; Görlacher et al., 2020). Indeed, glycosylation abnormalities are sometimes only detected during or shortly after episodes of fever (Reunert et al., 2019). Therefore, aggressive management of fever with antipyretics is recommended in order to preserve glycosylation capacities. Likewise, infections should be treated liberally.

Coagulation abnormalities both in the form of thrombotic events and impaired hemostasis are frequently seen in CDG. The underlying abnormalities are complex and in many cases, a somewhat fragile equilibrium seems to exist (Stibler et al., 1996). While thrombosis can be treated with low molecular weight heparin and also rivaroxaban (Lefrère et al., 2018; Altassan et al., 2019), impaired hemostasis and bleeding diathesis should be treated using fresh-frozen plasma rather than single factor substitution in order to avoid unwanted effects (Brucker et al., 2020).

#### Treating Ataxia and Stroke-Like Episodes in PMM2-CDG – Acetazolamide to the Rescue

Among the multitude of symptoms found in PMM2-CDG, ataxia accounts for a considerable burden of disease. The radiographic correlate is pronounced cerebellar ataxia, oftentimes diagnosed in other subtypes as well (Barone et al., 2014). Another complication of PMM2-CDG are so called stroke-like episodes (SLE) in which hemiparesis in the absence of any ischemic or hemorrhagic intra-cranial lesions is observed. There is data suggesting that SLE have an epileptic origin and they frequently improve following the administration of anticonvulsive medication (Dinopoulos et al., 2007). Recent research has identified gain-of-function effects of the Ca_*V*_2.1 voltage-gated calcium channel that are mediated by hypoglycosylation of both subunits α_1__*A*_ and α_2__*A*_ as a potential pathomechanism in SLE (Izquierdo-Serra et al., 2018).

Interestingly, dysfunction of Ca_*V*_2.1 caused by mutations in *CACNA1A* has been identified in Familial hemiplegic migraine 1 (FHM1; Ophoff et al., 1996), where altered channel kinetics were identified as a pathomechanism (Kraus et al., 1998, 2000). Other diseases associated with *CACNA1A* mutations are Spinocerebellar Ataxia type 6 (SCA6; Zhuchenko et al., 1997) and Developmental and Epileptic Encephalopathy 42 (Epi4K Consortium, 2016), which all show phenotypic similarities to the symptoms observed in PMM2-CDG.

The carbonic anhydrase inhibitor acetazolamide was shown to reduce cerebellar symptoms in SCA6 (Yabe et al., 2001) and FHM1 (Athwal and Lennox, 1996). It is believed that acetazolamide reduces overactivity, i.e., a gain of function, by altering the intracellular pH (Bain et al., 1992), making it a potential treatment for cerebellar symptoms in PMM2-CDG. The landmark randomized AZATAX trial showed substantial improvement of ataxia as assessed by International Cooperative Ataxia Rating Scale (ICARS) scores and general clinical improvement, while being generally well tolerated (Martínez-Monseny et al., 2019). Due to the limited observation period, no formal assessment of the effect on SLE frequency or severity could be made, although one patient who was frequently experiencing SLE did not do so during treatment.

#### Histone Deacetylase Inhibition Is a Targeted Therapy for PIGM-CDG

Inherited glucosylphosphatidylinositol (GPI) deficiency or PIGM-CDG is caused by a mutation in the core promoter of *PIGM*, severely impairing the binding site of the transcription factor Sp1 and resulting in hindered transcription (Almeida et al., 2006). In affected individuals, thrombotic events, seizures, and global hypotonia are present. Prompted by the lack of histone acetylation at the *PIGM* promoter, *in vitro* studies of the effect of the histone deacetylase inhibitor sodium butyrate indicated normalized Histone 4 acetylation and increased transcriptional activity as well as restored surface expression of GPI (Almeida et al., 2007). In a single patient trial of sodium butyrate at a dose of 20 mg/kg bodyweight three times a day, *PIGM* transcription and GPI expression increased *in vivo* as well, which was accompanied by dramatic clinical improvement with absence of seizures, returning of walking abilities and restored self-feeding (Almeida et al., 2007). Higher doses of 30 mg/kg three times a day were tolerated as well. Another study in three individuals carrying the same mutation found modest clinical improvement while not demonstrating increased GPI expression. However, these results were reported to have been hampered by incomplete compliance (Pode-Shakked et al., 2019).

#### Transplantation of Organs and Cells

As outlined above, mannose supplementation is not successful in preventing hepatic fibrosis in MPI-CDG. This has been reported to necessitate liver transplantation in female patient (Janssen et al., 2014). The patient showed profound clinical improvement in addition to normalization of biochemical parameters. Similarly, liver transplantation in CCDC115-CDG led to improvement in a patient although several individuals had died previously following repeated transplantations and associated complications (Jansen et al., 2016). While attempted in COG6-CDG (Rymen et al., 2015), no definite judgment on the efficacy in this subtype can be made since the patient died due to transplant associated complications.

Heart transplantation was attempted in DOLK-CDG in several cases (Kapusta et al., 2013; Klcovansky et al., 2016), showing favorable outcomes.

Due to the predominantly immunocompromised phenotype in PGM3-CDG, hematopoietic stem cell transplantation from bone marrow and cord blood was performed in two individuals, leading to correction of neutro- and lymphopenia (Stray-Pedersen et al., 2014).

#### Emerging Concepts

Given the successful application of antisense and gene therapy approaches in disorders such as spinal muscular atrophy and *RPE65*-mediated inherited retinal dystrophy (Finkel et al., 2017; Mendell et al., 2017; Russell et al., 2017; Day et al., 2021), similar approaches in CDG are being actively explored. However, none of the currently studied therapies has advanced to clinical or human *in vivo* application (Vega et al., 2009; Tal-Goldberg et al., 2014; Haenseler et al., 2018).

## Conclusion

Congenital disorders of glycosylation represent an ever-growing, complex family of disorders with a severe presentation in virtually all organ systems. Although treatment options for most subtypes are still lacking, recent years have seen substantial advances in the treatment of these ultra-rare diseases. Due to the rising numbers in patients, controlled trials now seem possible and have even been attempted, thus finally producing a robust scientific basis for clinical application. Further, collaborative efforts are needed to assure optimal treatment for patients with CDG.

## Author Contributions

JP and TM drafted, revised, and approved the final version of the article. Both authors contributed to the article and approved the submitted version.

## Conflict of Interest

The authors declare that the research was conducted in the absence of any commercial or financial relationships that could be construed as a potential conflict of interest.

## Publisher’s Note

All claims expressed in this article are solely those of the authors and do not necessarily represent those of their affiliated organizations, or those of the publisher, the editors and the reviewers. Any product that may be evaluated in this article, or claim that may be made by its manufacturer, is not guaranteed or endorsed by the publisher.
